# Sociodemographic, geographic and temporal patterns of snakebites in Brazil: a descriptive study, 2010-2022

**DOI:** 10.1590/S2237-96222026v35e20250181.en

**Published:** 2026-05-11

**Authors:** Gisele Dias de Freitas, Thiago Salomão de Azevedo, Anderson de Oliveira, Alec Brian Lacerda, Gabriella Pereira dos Santos, Etna Leal, Flávio Santos Dourado, Hui Wen Fan, Francisco Chiaravalloti-Neto

**Affiliations:** ¹ Secretaria da Saúde do Estado de São Paulo, Divisão de Zoonoses, São Paulo, SP, Brazil Secretaria da Saúde do Estado de São Paulo Divisão de Zoonoses São Paulo SP Brazil; 2 Universidade de São Paulo, Faculdade de Saúde Pública, São Paulo, SP, Brazil Universidade de São Paulo Faculdade de Saúde Pública São Paulo SP Brazil; 3 Universidade Estadual Paulista, Instituto de Biociências, Rio Claro, SP, Brazil Universidade Estadual Paulista Instituto de Biociências Rio Claro SP Brazil; 4 Ministério da Saúde, Coordenação-Geral de Vigilância de Zoonoses e Doenças de Transmissão Vetorial, Brasília, DF, Brazil Ministério da Saúde Coordenação-Geral de Vigilância de Zoonoses e Doenças de Transmissão Vetorial Brasília DF Brazil; 5 Instituto Butantan, Centro Bioindustrial, São Paulo, SP, Brazil Instituto Butantan Centro Bioindustrial São Paulo SP Brazil

**Keywords:** Snake Bites, Animals, Poisonous, Antivenins, Risk Areas, Descriptive Study., Mordeduras de Serpientes, Animales Ponzoñosos, Antivenenos, Áreas de Riesgo, Estudio Descriptivo.

## Abstract

**Objective:**

To analyze snakebite accidents that occurred in Brazil between 2010 and 2022, focusing on the sociodemographic characteristics of victims, snake genus involved, seasonality, geographic distribution, degree of risk and relationship with biomes. This descriptive study was based on data taken from the Notifiable Health Conditions Information System on accidents caused by *Bothrops*, *Crotalus*, *Lachesis* and *Elapidae*.

**Methods:**

Incidence rates, mortality rates and case fatality ratios were calculated according to snake genus, age and sex. Spatial analysis used Bayesian statistics and the Getis-Ord index (Gi*) to identify areas with the highest concentration of cases and classify regional risk (from very low to very high).

**Results:**

Between 2010 and 2022, 329,269 snakebite accidents were recorded in Brazil, with a predominance of bothropic cases (70.6%). In the same period, 1,514 deaths occurred, 74.2% of which were associated with bothropic snakebites, 20.7% with crotalic snakebits, 4.7% with lachetic snakebites and 0.4% with elapid snakebites. Despite their lower frequency, crotalic snakebites had the highest annual case fatality ratio (0.1%). Most victims were males (77.8%), of Brown skin color (58.4%), adults (76.3%), with low levels of schooling (48.0%), and living in rural areas (63.9%). Deaths were associated with delays in care. Bothropic snakebites remained stable, crotalic and elapid snakebites increased, while lachetic snakebites decreased. Cases occurred mostly between October and March, except in the Northeast, where they were mostly between December and June. Geographically, the patterns followed the biomes.

**Conclusion:**

The results reinforce the need for specific regional strategies for surveillance, prevention and effective management, based on the observed geographic, seasonal and demographic patterns.

## Resumen

**Objetivo:**

Analizar los accidentes por mordeduras de serpiente ocurridos en Brasil entre 2010 y 2022, centrándose en las características sociodemográficas de las víctimas, los géneros de serpientes involucradas, la estacionalidad, la distribución geográfica, el grado de riesgo y la relación con los biomas. Este estudio descriptivo se basó en datos del Sistema Nacional de Información de Enfermedades de Declaración Obligatoria sobre accidentes causados por *Bothrops*, *Crotalus*, *Lachesis* y *Elapidae*.

**Métodos:**

Se calcularon las tasas de incidencia, mortalidad y letalidad por género de serpiente, edad y sexo. El análisis espacial utilizó estadística bayesiana y el índice Getis-Ord (Gi*) para identificar las áreas con mayor concentración de casos y clasificar el riesgo regional (de muy bajo a muy alto).

**Resultados:**

Entre 2010 y 2022, se registraron 329.269 accidentes por mordeduras de serpiente en el país, con predominio de casos botrópicos (70,6%). En el mismo período se produjeron 1.514 muertes, de las cuales el 74,2% estuvieron asociadas a accidentes con *Bothrops*, el 20,7% con *Crotalus*, el 4,7% con *Lachesis* y el 0,4% con *Elapidae*. A pesar de su menor frecuencia, las mordeduras de serpientes crotálicas tuvieron la tasa de letalidad anual más alta (0,1%). La mayoría de las víctimas fueron hombres (77,8%), mestizos (58,4%), adultos (76,3%), con bajos niveles de educación (48,0%) y residentes en áreas rurales (63,9%). Las muertes se asociaron con retrasos en la atención. Las mordeduras de serpientes botrópicas se mantuvieron estables, las mordeduras de serpientes crotálicas y elápidas aumentaron, mientras que las mordeduras de serpientes del género *Lachesis* disminuyeron. Los casos ocurrieron principal­mente entre octubre y marzo, excepto en el noreste, entre diciembre y junio. Geográficamente, los patrones siguieron los biomas.

**Conclusión:**

Los resultados refuerzan la necesidad de estrategias regionales específicas para la vigilancia, prevención y gestión eficaz, basadas en los patrones geográficos, estacionales y demográficos observados.

**Ethical aspects**

This research used public domain anonymized databases.

## Introduction 

Snakebite envenoming is a neglected tropical disease that results in approximately 138,000 deaths worldwide annually, in addition to causing suffering and disability, especially in poor communities in low- and middle-income countries ^1^. Recognizing its seriousness, in 2017 the World Health Organization included snakebite envenoming on the list of neglected tropical diseases and, in 2019, approved the *Strategy for a globally coordinated response to a priority neglected tropical disease*, which established the goal of reducing snakebite-related deaths and disabilities by 50% by 2030 through global efforts. The main strategies are: (i) improving surveillance of the disease; (ii) increasing epidemiological knowledge of envenoming to identify and serve more effectively populations requiring greater preventive education, appropriate treatment and long-term care due to sequelae; and (iii) expanding the availability, accessibility and cost reduction of antivenins globally to ensure safe and effective pharmacotherapeutic treatments for those injured ^2^^,^^3^. 

In Brazil, due to its vast biodiversity, accidents involving poisonous animals are highly significant for public health. In the period analyzed, Brazil ranked third worldwide in terms of the number of snakebites, alongside Vietnam following India and Sri Lanka (4.5%). In 2023, 32,595 accidents and 143 deaths were recorded in Brazil, mainly in the country's North and Northeast regions, which together accounted for almost two-thirds of cases, despite being home to only 36% of the Brazilian population. These accidents are exacerbated by factors such as the invasion of natural habitats and the accumulation of garbage and debris, which attract small animals, which in turn are food for snakes ^6^.

Since the creation of antivenin by Vital Brazil and the founding of the Butantan Institute in 1901 ^7^ and the implementation of the National Snakebite Program in 1986, following a major crisis in serum production in the country that culminated in the death of a child in Brasília ^8^, Brazil has invested in controlling accidents involving poisonous animals. Reports of these accidents are recorded on the Notifiable Health Conditions Information System (*Sistema de Informação de Agravos de Notificação* - SINAN) and have become more representative, especially since 2010, after the disease was included on the mandatory reporting list ^9^. At that time, this information provided fundamental data dor epidemiological surveillance and public health action planning.

In Brazil, snakes of the Viperidae family (lanceheads, rattlesnakes and bushmasters) and Elapidae family (coral snakes) are responsible for bothropic, crotalic, lachetic and elapid accidents, respectively ^10^. Among them, the *Bothrops* genus is responsible for the majority of accidents, while the *Crotalus* genus has the highest case fatality ratio ^11^.

Treatment for poisoning after an accident requires administration of antivenin (serum) specific to each type of accident. These serums are provided exclusively by the Ministry of Health, forming part of the Strategic Component of Pharmaceutical Care, and are included on the National List of Essential Medicines. These immunobiologicals are available free of charge only from public health facilities, which are strategic points designated by the Brazilian National Health System to serve the population. The Ministry of Health's Health Surveillance Guide describes treatment protocols, including the number of vials to be used according to the severity of the accident (mild, moderate, or severe) ^12^.

Antivenins are biological products obtained from the processed and purified plasma of horses previously immunized with animal venom antigens. Their manufacturing process is time-consuming and dependent on the animals' immune response, making their use restricted to cases of absolute necessity. To minimize complications and reduce the risk of sequelae, treatment should be administered as quickly as possible, ideally within the first six hours after the bite ^12^.

Given this scenario, the main objective of this study was to analyze snakebites that occurred in Brazil between 2010 and 2022, focusing on the sociodemographic characteristics of the victims, the genus of the snakes involved, seasonality, geographic distribution, degree of risk and relationship with the country's biomes.

The results obtained aim to contribute to the identification of areas of greatest risk, supporting prevention strategies, allocation of resources in priority regions and adequate planning of care and distribution of serums, according to the type of accident and regional patterns.

## Methods 

Study type and data source

A descriptive study was conducted on snakebite accidents that occurred in Brazil between 2010 and 2022. Data were extracted from the SINAN, selecting all reports of accidents with poisonous animals involving snakes of the *Bothrops*, *Crotalus* and *Lachesis* genera, and the *Elapidae* family. Only records with complete data on the type of snake and the location of the accident were included. An autochthonous accident was considered one in which the patient's municipality of residence coincided with the municipality in which the accident occurred.

Contextualization

The variables analyzed included sociodemographic characteristics of the injured individuals (age, sex, race/skin color and schooling), as well as spatiotemporal information (date of accident, municipality of residence and occurrence, area of ​​occurrence), and variables related to medical care and outcome (time between accident and care, as well as clinical progression). Additional demographic and geographic information was obtained from the Brazilian Institute of Geography and Statistics, including data on the Brazilian biomes (Amazon, *Caatinga*, Savanna (*Cerrado*), Atlantic Forest, Pampas and Wetland (*Pantanal*)) ^13^.

Statistical analysis

Incidence and mortality rates and case fatality ratios were calculated by snake genus, age group (0-9 years, 10-19 years, 20-59 years and ≥60 years) and sex. The annual incidence rate was obtained by dividing the average number of accidents by the estimated population for 2016 divided by 13 (the number of years of the study), then multiplying by 100,000 inhabitants. The mortality rate was calculated in the same way, replacing the number of accidents with the number of deaths. The case fatality ratio was determined by the ratio of the number of deaths to the total number of accidents, multiplied by 100,000 inhabitants.

With regard to the temporal analysis, the number of accidents was analyzed according to snake genus, geographic region, year of occurrence (temporality) and month of occurrence (seasonality). Incidence rate spatial distribution was analyzed using rates adjusted by local empirical Bayesian estimates ^14^, considering only autochthonous accidents by snake type and municipality of occurrence. The Getis-Ord index (Gi*) was applied to identify areas of case concentration, which is capable of detecting spatial clusters with values ​​above or below the regional average, in addition to quantifying discrepancies ^15^.

The annual occurrence count was normalized using Z-scores and organized per year by the maximum value within the geographic limit ^16^. Risk stratification was performed based on the percentage frequency of municipalities identified as areas of case concentration throughout the study period, classified into five levels: very low (≤20%), low (20%-40%), medium (40%-60%), high (60%-80%) and very high (>80%). The analyses were performed using Excel, GeoDa, QGIS, R (version 4.3.2), and Tabwin software. The results were presented by means of qualitative maps separated by snake genus, and this database can be accessed on the Zonedo platform ^17^.

## Results 

Between 2010 and 2022, 329,269 accidents caused by venomous snakes were reported in Brazil. Of this total, 283,245 (70.6%) were bothropic, 33,447 (8.3%) crotalic, 8,861 (2.2%) lachetic and 3,716 (0.9%) elapid. In the same period, 1,514 deaths related to these accidents were recorded, of which 1,123 (74.2%) were attributed to bothropic snakebites, 314 (20.7%) to crotalic snakebites, 71 (4.7%) to lachetic snakebites and 6 (0.4%) to elapid snakebites. Analysis of snakebite autochthony revealed that part of the cases occurred outside the municipality of residence, namely: 7.3% of bothropic snakebites, 9.6% of crotalic snakebites, 7.7% of elapid snakebites and 5.0% of lachetic snakebites.

Snakebites and deaths were most prevalent among people of Brown skin color (Brazilian mixed race), but a high proportion of Indigenous people was found regarding lachetic accidents (9.6% of accidents and 25.4% of deaths). Snakebite accidents were more frequent among males (77.8%), those of Brown skin color (58.4%), adults between 20 and 59 years of age (76.3%) and people who had only elementary education (48.0%). The majority occurred in rural areas (63.9%), although crotalic and elapid accidents had significant proportions in urban areas (42.2% and 46.6%). Regarding time to treatment, 65.3% of victims of bothropic, crotalic and elapid snakebites received treatment within three hours, while this percentage was lower for lachetic snakebites (44.1%), with 35.4% receiving treatment between three and 12 hours (Supplementary Table 1.

Regarding our sociodemographic assessment, the analysis of the educational attainment of snakebite victims by region revealed a predominance of individuals with low schooling, especially in the North, Northeast and Midwest regions. Illiteracy was more prevalent in the North and Northeast regions, while the South and Southeast revealed had lower illiteracy rates. Higher education, whether complete or incomplete, was low in all regions. However, the high percentage of records with unknown schooling level standed out: 28.4% in the North, 40.7% in the Northeast, 33.2% in the Midwest, 26.6% in the South and 39.4% in the Southeast. This compromised data quality and limited more accurate analyses (Supplementary Table 2.

The socioeconomic profile of fatalities was similar to that of the injured, but there was a higher proportion of Indigenous victims among Elapid (16.7%) and Lachetic (25.4%) cases. Low schooling levels (51.1%) were more common among fatalities, and the predominance of snakebites in rural areas was even more pronounced (78.9%). Time to treatment was significantly longer in fatal cases, especially for lachetic snakebites.

Bothropic snakebites were the most frequent in Brazil, with an incidence rate 7.4 times higher than crotalic snakebites, 77.5 times higher than elapid snakebites and 30.9 times higher than lachetic snakebites. Males were the most affected for all types of snakebites, particularly lachetic and crotalic snakebites, whereby incidence was three times higher than among females, while for elapid snakebites incidence among males was 1.5 times higher. Crotalic snakebites had the highest case fatality ratio, followed by lachetic snakebites, bothropic snakebites and elapid snakebites. Elderly individuals were the most likely to die after snakebites. The case fatality ratio varied regionally, being higher in the North and Midwest regions for bothropic, crotalic and lachetic snakebites, and in the Southern region for crotalic and elapid snakebites. Regarding age group, bothropic accidents were most common among 10-19 year-olds and 20-59 year-olds, with 12.0 and 11.4 cases per 100,000 population per year. Crotalic snakebites affected adults and the elderly the most, while elapid and lachetic snakebites followed the bothropic snakebite pattern Table 1.

**Table 1:** Analysis of snakebite accidents by sex, age group, region and type of snakebite, according to number (No.) of snakebites, number (No.) of deaths, incidence rates (100,00 inhabitants/year), mortality rates (100,000 inhabitants/year) and case fatality ratio (%). Brazil, 2010-2022 (n=329,269)

Type of snakebite	Variable	Sex^a^		>Age group (years)				Region of Brazil^b^					Total
		F	M	0-9	10-19	20-59	≥60	N	NE	CO	SE	S	
Bothropic	No. snakebites	63,511	219,734	20,697	47,069	180,862	34,617	106,926	63,210	30,223	59,619	23,267	283,245
	Incidence rate	4.48	16.21	5.42	12.00	11.36	8.50	43.50	8.43	13.92	5.12	5.89	10.21
	No. deaths	309	814	65	82	571	405	414	328	147	166	68	1.123
	Mortality rate	2.18	6.01	1.70	2.09	3.59	9.94	16.84	4.38	6.77	1.42	1.72	4.05
	Case fatality ratio	0.37	0.28	0.24	0.13	0.24	0.90	0.03	0.04	0.04	0.02	0.02	0.03
Crotalic	No. snakebites	6,825	26,622	2,402	4,450	21,707	4,888	2,949	12,535	4,580	11,797	1,586	33,447
	Incidence rate	0.48	1.96	0.63	1.13	1.36	1.20	1.20	1.67	2.11	1.01	0.40	1.21
	No. deaths	64	250	21	25	149	119	38	163	31	68	14	314
	Mortality rate	0.45	1.84	0.55	0.64	0.94	2.92	1.55	2.17	1.43	0.58	0.35	1.13
	Case fatality ratio	0.72	0.72	0.67	0.43	0.53	1.87	0.10	0.10	0.05	0.04	0.07	0.07
Lachetic	No. snakebites	1,666	7,195	754	1,849	5,495	763	7,956	495	291	101	18	8,861
	Incidence rate	0.12	0.53	0.20	0.47	0.35	0.19	3.24	0.07	0.13	0.01	0.00	0.32
	No. deaths	13	58	11	6	35	19	68	0	3	0	0	71
	Mortality rate	0.09	0.43	0.29	0.15	0.22	0.47	2.77	0.00	0.14	0.00	0.00	0.06
	Case fatality ratio	0.60	0.62	1.12	0.25	0.49	1.92	0.07	0.00	0.08	0.00	0.00	0.06
Elapid	No. snakebites	1,080	2,636	353	530	2,483	350	437	2,043	270	741	225	3,716
	Incidence rate	0.08	0.19	0.09	0.14	0.16	0.09	0.18	0.27	0.12	0.06	0.06	0.13
	No. deaths	1	5	0	1	3	2	1	3	0	1	1	6
	Mortality rate	0.01	0.04	0.00	0.03	0.02	0.05	0.04	0.04	0.00	0.01	0.03	0.02
	Case fatality ratio	0.07	0.15	0.00	0.15	0.09	0.44	0.02	0.01	0.00	0.01	0.03	0.01
Total	No. snakebites	73,082	256,187	24,206	53,898	210,547	40,618	118,268	78,283	35,364	72,258	25,096	329,269
	Incidence rate	5.51	20.12	6.25	12.23	14.32	13.44	48.12	10.44	16.28	6.20	6.35	12.66
	No. deaths	387	1,127	97	114	758	545	521	494	181	235	83	1.514
	Mortality rate	0.03	0.09	0.03	0.03	0.05	0.18	0.21	0.07	0.08	0.02	0.02	0.06
	Case fatality ratio	0.53	0.44	0.40	0.21	0.36	1.34	0.44	0.63	0.51	0.33	0.33	0.46

a Sex: F=female/M=male; ^b^Region: N=North, NE=Northeast, MW=Midwest, SE=Southeast, S=South.

Regarding regional distribution, bothropic snakebites were more prevalent in the North and less prevalent in the Southeast. Crotalic snakebite incidence was higher in the Midwest and lower in the South. Elapid snakebites were more frequent in the Northeast and, similarly, less frequent in the South and Southeast. Lachetic snakebites predominated in the North and were less frequent in the Southeast. In the South, no reports of lachetic snakebites were expected, as there are no records of snakes of this genus in the wild in that region Table 1 ^18^.

Snakebite accidents showed different temporal trends during the period assessed. Bothropic snakebites maintained a stable occurrence pattern in the Midwest region, with an increasing number of snakebites in the North and Northeast regions and a decreasing number in the South and Southeast regions. Crotalic and elapid snakebites showed an increasing trend, with effect from 2013, in the South and Northeast regions, and a stable trend in the other regions. Lachetic snakebites, on the other hand, showed a sharp and continuous reduction in the number of cases in the Northern region from 2010 onwards Figures 2A to 2D).

**Figure 1:**
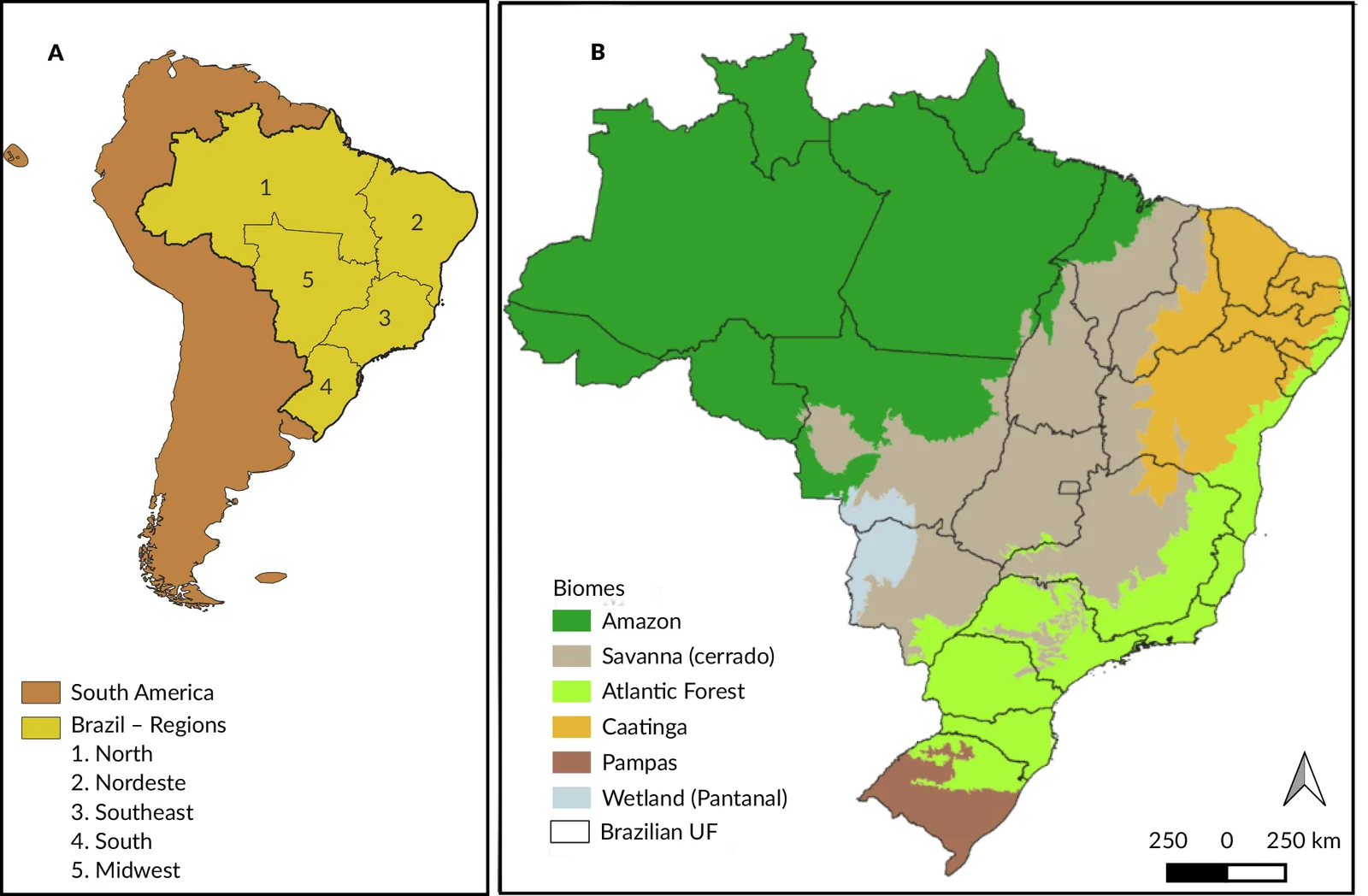
Geospatial view of South America with details of the Brazilian territory: (A) regions; (B) biomes and federative units (unidades federativas - UF)

**Figure 2:**
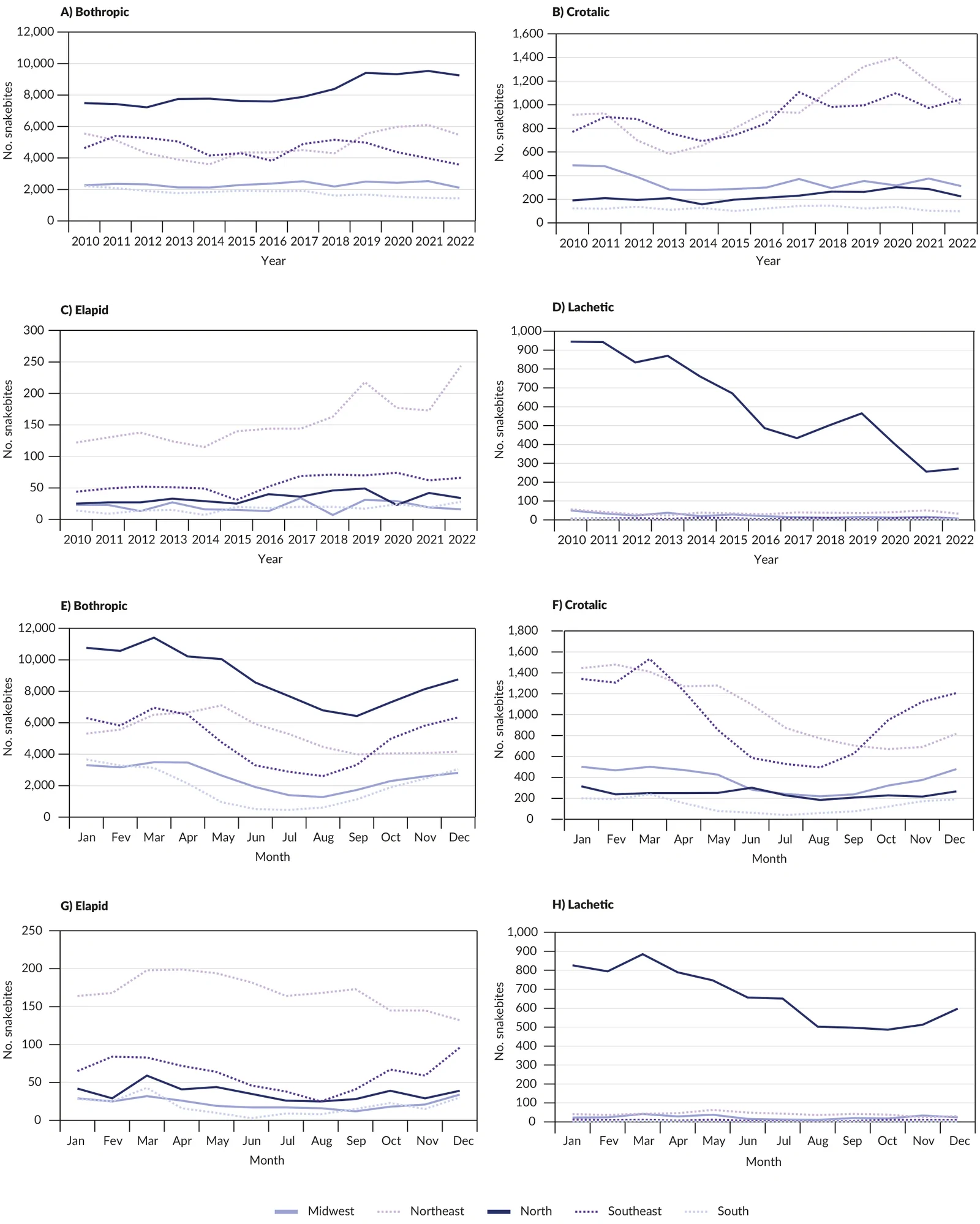
Temporal analysis (A-D) and seasonal temporal analysis (E-H) of snakebites stratified by type of snakebite and by region. Brazil, 2010-2022 (n=329,269)

In the seasonality analysis, taking the month of occurrence, distinct regional patterns emerged. In the case of bothropic snakebites, the Midwest, Northern, Southeast and Southern regions had the highest frequency between October and March, while in the Northeast region, the period of highest occurrence was between February and June, in contrast to the other regions. In the case of crotalic snakebites, the Midwest, Southeast and Southern regions followed a similar pattern, with the highest number of cases between November and April, while in the Northern and Northeast regions, seasonality was more pronounced between December and June. Elapid snakebites showed a uniform pattern across regions, with the highest occurrence of cases between January and May. With regard to lachetic snakebites, the Northern region stood out, with an increase in cases starting in December, peaking in March, and the lowest number of occurrences between August and September Figures 2E to 2H).

Regardless of the genus of snake that caused the accident, there was a strong correlation between the spatial patterns of distribution of annual incidence rates, locations of greatest risk and locations where deaths occurred after the aforementioned accidents Figures 3 and 4).

**Figure 3:**
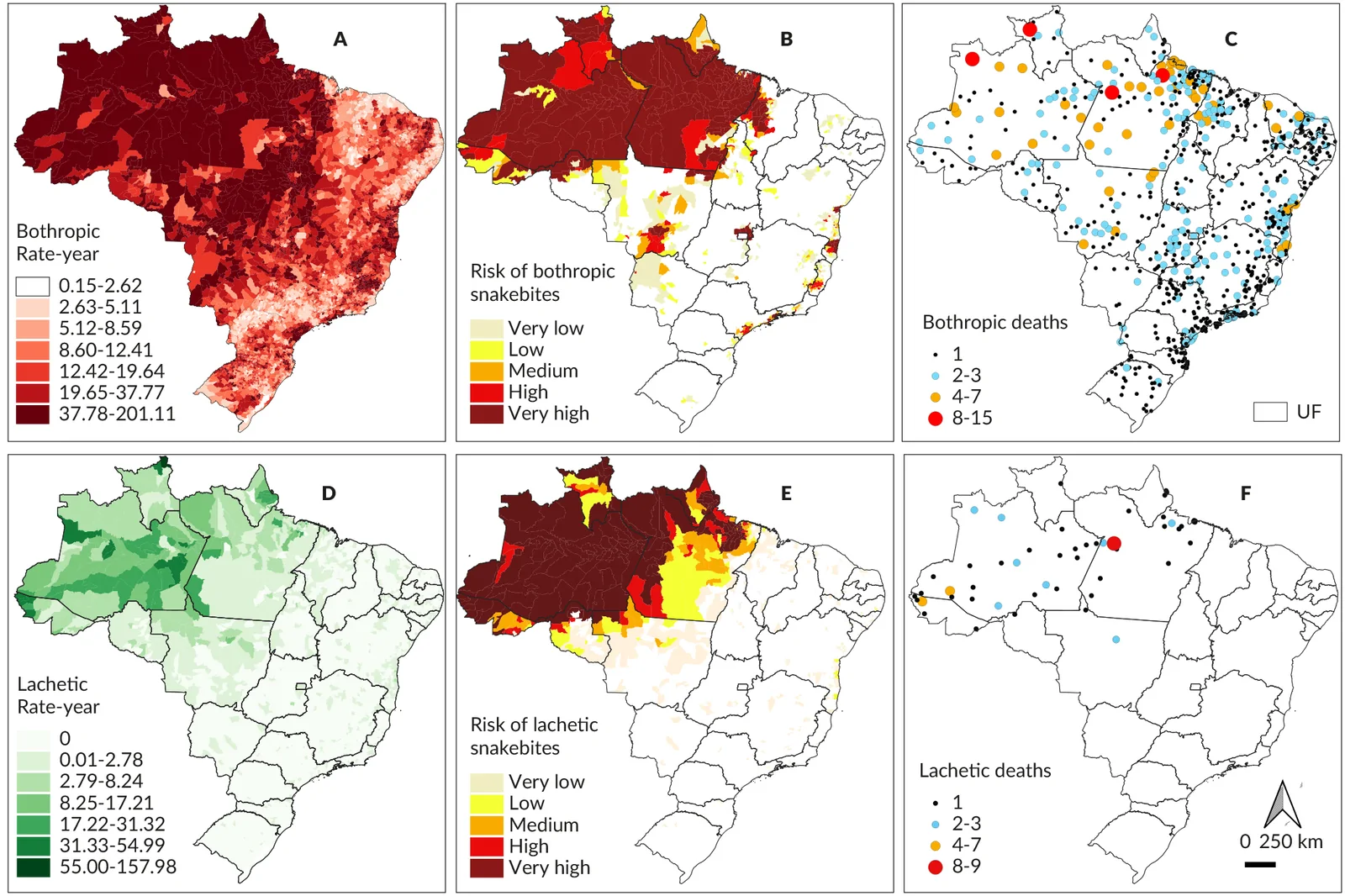
Spatial distribution of bothropic snakebites (n=283,245) (A-C) and lachetic snakebites (n=8,861) (D-F) by municipalities and states stratified by Bayesian incidence rates^a^ (A and D), risk areas (B and E) and number of deaths (C and F). Brazil, 2010-2022

Bothropic snakebites were widely distributed throughout Brazil. However, as shown in Figure 3A, which can also be related to the biome distribution in Figure 1, the regions with the highest annual bothropic snakebite incidence rates were found in the Amazon, Atlantic Forest and part of the Savanna (*Cerrado*) biome. This also coincided with the risk map for this type of snakebite and the highest occurrence of deaths Figures 3A to 3C). Lachetic snakebites presented a more characteristic and restricted distribution pattern, being more frequent in the Northern region and at the northernmost part of the Atlantic Forest biome, between the states of the Northeast region and the state of Espírito Santo Figure 3D). Crotalic snakebites, on the other hand, presented a distribution pattern in which the Savanna (*Cerrado*) biome predominated, as well as in the states that are at the northernmost part of the Amazon biome (states of Amapá and Roraima) Figure 4A). Finally, in relation to elapid snakebites, locations with a higher risk of accidents were found in the Amazon, Savanna (*Cerrado*), *Caatinga* and Atlantic Forest biomes Figure 4D).

**Figure 4:**
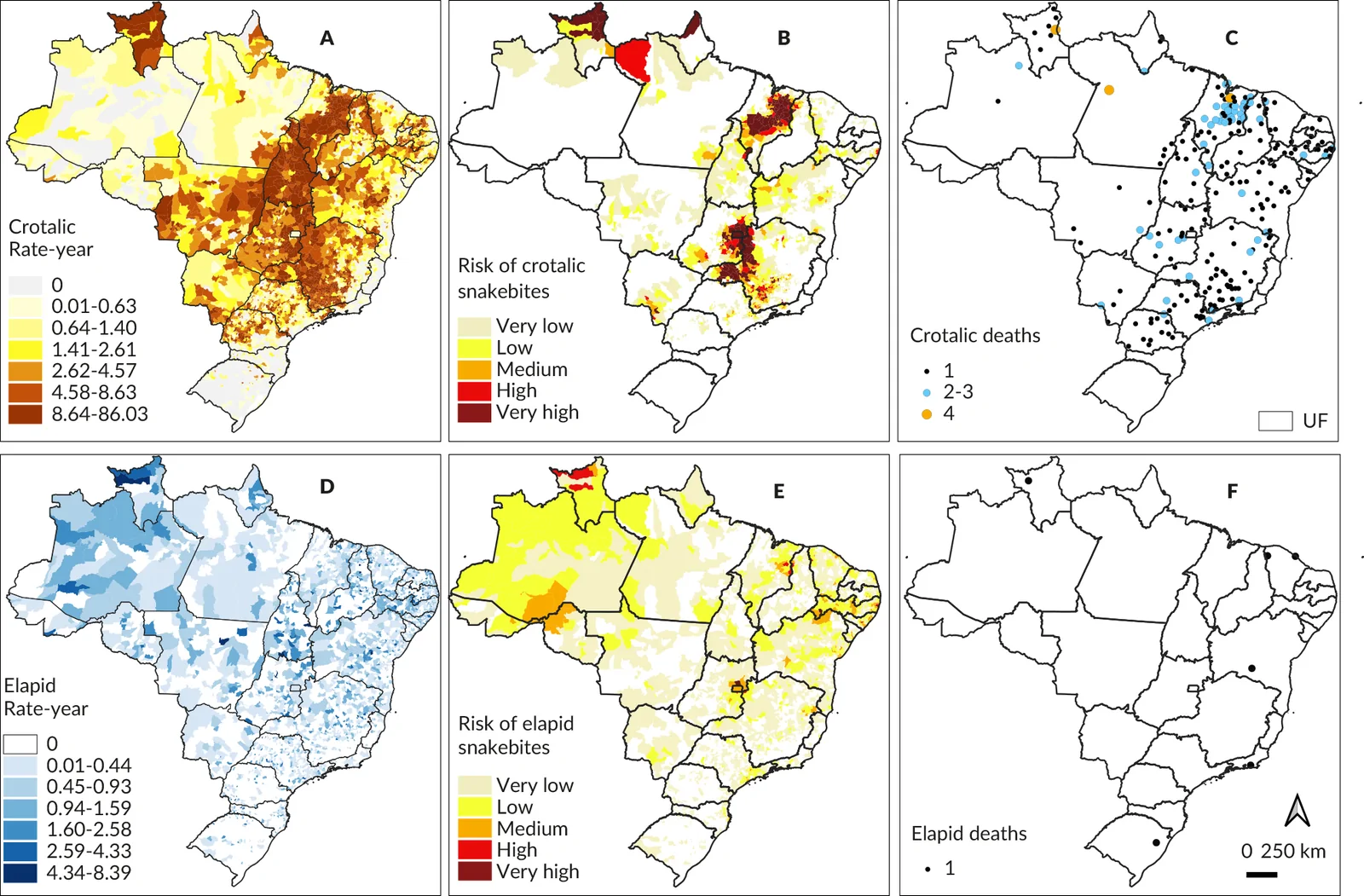
Spatial distribution of crotalic snakebites (n=33,447) (A-C) and elapid snakebites (n=3,716) (D-F) by municipalities and states stratified by Bayesian incidence rates^a^ (A and D), risk areas (B and E) and number of deaths (C and F). Brazil 2010-2022

## Discussion 

Between 2010 and 2022, snakebite accidents in Brazil showed striking patterns regarding geographic distribution, victim profile and clinical characteristics. Bothropic snakebites predominated, accounting for two-thirds of cases and deaths, which may reflect the widespread distribution and adaptability of snakes of the *Bothrops* genus. Although less frequent, crotalic snakebites had a higher case-fatality ratio, highlighting their clinical severity. Most victims were adult men, of Brown skin color, with low schooling levels and living in rural areas, indicating a relationship between socioeconomic vulnerability, occupational exposure and limited access to health care. We also found that lachetic snakebites, although declining, remain relevant due to their high case-fatality ratio and their occurrence in remote regions, such as the Amazon. Case seasonality varied regionally, with a higher concentration during the rainy season, suggesting the influence of ecological and climatic factors on snake activity and the risk of accidents.

Among the limitations of this study is its reliance on secondary data from the Notifiable Health Conditions Information System, which is subject to underreporting, inconsistencies and incompleteness. Furthermore, errors in identifying snakes or the type of envenomation can lead to data distortions, such as possible overestimation of lachetic snkaebites due to indistinct common nomenclature in some regions. Another limitation is the lack of detailed data on the location of deaths and the differentiation between urban and rural areas, which compromises more refined analyses of risk factors and access to health services. Furthermore, the low completeness of variables related to the snakebite victims' educational level limits a more detailed analysis of the victims' sociodemographic profile.

The high frequency of bothropic snakebites is consistent with previous studies highlighting the wide distribution of snakes of the *Bothrops* genus across all Brazilian biomes, from dense forests to anthropized areas ^11^^,^^19^. This ecological plasticity favors contact with humans in different contexts. The high case fatality ratio of crotalic snakebites can be attributed to the toxicity of the venom and the rapid progression of clinical symptoms, requiring early diagnosis and immediate administration of antivenin, as also observed in a retrospective cohort study conducted in the Midwest region ^20^. Regarding lachetic snakebites, in addition to the severity of envenomation, geographic and cultural factors hinder access to health care, especially among Indigenous populations, who sometimes initially turn to traditional medicine. This cultural barrier was also observed in qualitative research conducted with Amazonian communities ^21^^,^^22^.

The sociodemographic profile of the victims is consistent with findings from other cross-sectional studies conducted in rural areas of the Northeast and Northern regions of Brazil, which indicate a predominance of accidents among young adults, rural workers and men ^9^^,^^23^. This suggests that outdoor economic activities are directly related to risk exposure. The higher frequency of deaths among Indigenous people, especially due to lachetic snakebites, can be explained by both geographic isolation and limited resources in local health services, as already described in investigations conducted in the Brazilian Legal Amazon ^24^.

The temporal analysis revealed the stability of bothropic snakebites, probably associated with the widespread distribution of the genus and constant contact with humans. In contrast, the increase in crotalic snakebites may be related to the expansion of these species into degraded areas, as described in studies on land use change and climate change ^11^^,^^19^^,^^25^. The reduction in lachetic cases, in turn, may result from the reduction in snake habitats and improved reporting systems. The seasonality observed coincides with the period of greatest snake activity and increased agricultural practices ^26^^,^^27^.

The spatial analysis revealed a higher case fatality ratio for snakebites recorded in the North and Midwest regions, consistent with previous studies that associated mortality with prolonged time lapses between accident and medical care, reflecting low health service coverage in remote regions ^23^. The presence of reports of lachetic snakebites outside the species' habitual area reinforces the hypothesis of misidentification or confusion in regional nomenclature, as occurs in the Alto Juruá region, where *Bothrops atrox* is called "*surucucu*", this being the same name given to *Lachesis muta*^28^^,^^29^. Errors such as this compromise data accuracy and require investment in education and training for health professionals responsible for reporting cases.

The occurrence of non-autochthonous accidents emphasizes the mobility of victims and the logistical challenges of providing rapid care. Strategies such as the São Paulo State Health Department's online tool for locating units with antivenin demonstrate potential for mitigating delays and should be replicated in other regions ^30^. Standardizing regional nomenclature and training health care professionals can significantly contribute to improving the quality of reporting and health care responses.

In summary, the findings of this study reveal the complexity of snakebite accidents in Brazil, which are strongly influenced by ecological, climatic, geographic and social factors. The predominance of bothropic snakebites, the high case fatality ratio of crotalic and lachetic snakebites, as well as unequal access to health services, highlight the need for specific regional strategies for snakebite surveillance, prevention and management. Furthermore, the importance of actions aimed at improving the quality of data recorded on the Notifiable Health Conditions Information System is highlighted, in order to allow for more consistent and in-depth analyses of the victim profile. The results obtained in this study provide support for more effective and equitable public policies, especially targeted at vulnerable populations and hard-to-reach regions, promoting an integrated approach between health, the environment and animal management.

## Data Availability

The database, codes, methods and other materials used and resulting from the research used in this study are available for consultation on the Zenodo platform. Available in: Freitas GD de, Oliveira A de. Acidentes ofídicos no Brasil (2010-2022): um retrato nacional dos padrões sociodemográficos, geográficos e temporais em estudo descritivo [Internet]. Zenodo; 2025. Available from: https://doi.org/10.5281/zenodo.15571022.
