# Supplementary material for: Sociodemographic, geographic and temporal patterns of snakebites in Brazil: a descriptive study, 2010-2022
Source: Epidemiol Serv Saude. 2026 May 11;35:e20250181. doi: 10.1590/S2237-96222026v35e20250181.en (PMC13686398; doi:10.1590/S2237-96222026v35e20250181.en)
Supplement: Supplementary file 1 [file 2237-9622-ress-35-e20250181-tabelas-suplementares-en.pdf]

Supplementary Table 1. Sociodemographic characteristics of snakebites in Brazil between 2010 and 2022 (n=329,269): analysis by race/skin color, level of schooling, area in which snakebite occurred and time lapse between snakebite and treatment. Data presented in absolute numbers (N) and percentages (%) of cases and deaths, according to type of snakebite

	Sociodemographic Assessment									
Race/skin color	Bothropic		Crotalic		Elapid		Lachetic		Total	
	Cases	Deaths	Cases	Deaths	Cases	Deaths	Cases	Deaths	Cases	Deaths
	N (%)	N (%)	N (%)	N (%)	N (%)	N (%)	N (%)	N (%)	N (%)	N (%)
Brown	163,246 (57.63)	640 (56.99)	17,722 (52.99)	195 (62.10)	2,089 (56.22)	2 (33.33)	6,569 (74.13)	47 (66.20)	189,626 (57.59)	884 (58.39)
White	63,502 (22.42)	219 (19.50)	9,107 (27.23)	54 (17.20)	748 (20.13)	1 (16.67)	487 (5.50)	3 (4.23)	73,844 (22.43)	277 (18.30)
Black	21,460 (7.58)	82 (7.30)	2,680 (8.01)	20 (6.37)	263 (7.08)	0 (0.00)	502 (5.67)	1 (1.41)	24,905 (7.56)	103 (6.80)
Indigenous	12,445 (4.39)	96 (8.55)	958 (2.86)	17 (5.41)	81 (2.18)	1 (16.67)	847 (9.56)	18 (25.35)	14,331 (4.35)	132 (8.72)
Asian	2,310 (0.82)	5 (0.45)	282 (0.84)	2 (0.64)	17 (0.46)	0 (0.00)	48 (0.54)	0 (0.00)	2,657 (0.81)	7 (0.46)
Unknown	20,282 (7.16)	81 (7.21)	2,698 (8.07)	26 (8.28)	518 (13.94)	2 (33.33)	408 (4.60)	2 (2.82)	23,906 (7.26)	111 (7.33)
Total	283,245 (100.00)	1,123 (100.00)	33,447 (100.00)	314 (100.00)	3,716 (100.00)	6 (100.00)	8,861 (100.00)	71 (100.00)	329,269 (100.00)	1,514 (100.00)
**Schooling**										
Illiterate	13,055 (4.61)	117 (10.42)	1,557 (4.66)	27 (8.60)	164 (4.41)	2 (33.33)	513 (5.79)	9 (12.68)	15,289 (4.64)	155 (10.24)
Elementary I	68,262 (24.10)	306 (27.25)	7,484 (22.38)	81 (25.80)	566 (15.23)	0 (0.00)	2,405 (27.14)	18 (25.35)	78,717 (23.91)	405 (26.75)
Elementary II	55,736 (19.68)	161 (14.34)	6,229 (18.62)	41 (13.06)	609 (16.39)	0 (0.00)	1,759 (19.85)	12 (16.90)	64,333 (19.54)	214 (14.13)
High school	33,121 (11.69)	62 (5.52)	4,264 (12.75)	16 (5.10)	472 (12.70)	0 (0.00)	873 (9.85)	3 (4.23)	38,730 (11.76)	81 (5.35)
Higher education	3,782 (1.34)	12 (1.07)	490 (1.47)	2 (0.64)	78 (2.10)	0 (0.00)	61 (0.69)	1 (1.41)	4,411 (1.34)	15 (0.99)
Not applicable	12,095 (4.27)	38 (3.38)	1,512 (4.52)	11 (3.50)	268 (7.21)	0 (0.00)	386 (4.36)	4 (5.63)	14,261 (4.33)	53 (3.50)
Unknown	97,194 (34.31)	427 (38.02)	11,911 (35.61)	136 (43.31)	1,559 (41.95)	4 (66.67)	2,864 (32.32)	24 (33.80)	113,528 (34.48)	591 (39.04)
Total	283,245 (100.00)	1,123 (100.00)	33,447 (100.00)	314 (100.00)	3,716 (100.00)	6 (100.00)	8,861 (100.00)	71 (100.00)	329,269 (100.00)	1,514 (100.00)

Supplementary Table 1. Continued

	Sociodemographic Assessment									
Race/skin color	Bothropic		Crotalic		Elapid		Lachetic		Total	
	Cases	Deaths	Cases	Deaths	Cases	Deaths	Cases	Deaths	Cases	Deaths
	N (%)	N (%)	N (%)	N (%)	N (%)	N (%)	N (%)	N (%)	N (%)	N (%)
**Area of Occurrence**										
Rural	177,568 (62.69)	724 (64.47)	17,928 (53.60)	183 (58.28)	1,774 (47.74)	4 (66.67)	6,565 (74.09)	56 (78.87)	203,835 (61.91)	967 (63.87)
Urbana	93,311 (32.94)	358 (31.88)	14,123 (42.23)	111 (35.35)	1,732 (46.61)	1 (16.67)	2,004 (22.62)	15 (21.13)	111,170 (33.76)	485 (32.03)
Peri-urban	2,664 (0.94)	3 (0.27)	215 (0.64)	0 (0.00)	56 (1.51)	0 (0.00)	56 (0.63)	0 (0.00)	2,991 (0.91)	3 (0.20)
Unknown	9,702 (3.43)	38 (3.38)	1,181 (3.53)	20 (6.37)	154 (4.14)	1 (16.67)	236 (2.66)	0 (0.00)	11,273 (3.42)	59 (3.90)
Total	283,245 (100.00)	1,123 (100.00)	33,447 (100.00)	314 (100.00)	3,716 (100.00)	6 (100.00)	8,861 (100.00)	71 (100.00)	329,269 (100.00)	1,514 (100.00)
**Time between Snakebite and Treatment**										
1-3h	95,272 (33.64)	283 (25.20)	10,894 (32.57)	74 (23.57)	1,125 (30.27)	1 (16.67)	2,453 (27.68)	6 (8.45)	109,744 (33.33)	364 (24.04)
0-1h	81,505 (28.78)	201 (17.90	11,617 (34.73)	48 (15.29)	1,323 (35.60)	1 (16.67)	1,452 (16.39)	6 (8.45)	95,897 (29.12)	256 (16.91)
3-6h	47,938 (16.92)	200 (17.81)	4,807 (14.37)	48 (15.29)	524 (14.10)	3 (50.00)	2,030 (22.91)	10 (14.08)	55,299 (16.79)	261 (17.24)
6-12h	19,403 (6.85)	105 (9.35)	1,720 (5.14)	36 (11.46)	191 (5.14)	0 (0.00)	1,112 (12.55)	17 (23.94)	22,426 (6.81)	158 (10.44)
12-24h	12,334 (4.35)	111 (9.88)	1,322 (3.95)	42 (13.38)	116 (3.2)	0 (0.00)	818 (9.23)	10 (14.08)	14,590 (4.43)	163 (10.77)
24h or more	10,894 (3.85)	125 (11.13)	1,070 (3.20)	36 (11.46)	104 (2.80)	0 (0.00)	557 (6.29)	12 (16.90)	12,625 (3.83)	173 (11.43)
Unknown	15,899 (5.61)	98 (8.73)	2,017 (6.03)	30 (9.55)	333 (8.96)	1 (16.67)	439 (4.95)	10 (14.08)	18,688 (5.68)	139 (9.18)
Total	283,245 (100.00)	1,123 (100.00)	33,447 (100.00)	314 (100.00)	3,716 (100.00)	6 (100.00)	8,861 (100.00)	71 (100.00)	329,269 (100.00)	1,514 (100.00)

Supplementary Table 2. Sociodemographic characteristics of snakebites in Brazil between 2010 and 2022 (n=329,269): analysis by level of schooling and region. Data presented in absolute numbers (N) and percentages (%)

Schooling	Midwest	Northeast	North	Southeast	South	Total
	N (%)	N (%)	N (%)	N (%)	N (%)	N (%)
Illiterate	1,148 (2.92)	5,257 (5.93)	6,483 (4.88)	1,637 (1.99)	396 (1.40)	14,921 (4.86)
Incomplete 1st to 4th grade - elementary	4,805 (12.22)	12,499 (14.11)	23,268 (17.51)	9,823 (11.95)	4,062 (14.38)	54,457 (17.74)
Complete 4th grade - elementary	2,105 (5.35)	4,721 (5.33)	8,646 (6.51)	5,027 (6.11)	2,364 (8.37)	22,863 (7.45)
Incomplete 5th to 8th grade - elementary	5,292 (13.46)	8,843 (9.98)	19,864 (14.95)	8,838 (10.75)	4,334 (15.34)	47,171 (15.36)
Complete elementary education	1,902 (4.84)	2,893 (3.26)	5,312 (4.00)	4,159 (5.06)	1,798 (6.37)	16,064 (5.23)
Incomplete high school education	2,211 (5.52)	3,047 (3.44)	6,080 (4.58)	3,980 (4.84)	1,495 (5.29)	16,813 (5.48)
Complete high school education	2,810 (7.15)	3,549 (4.01)	6,289 (4.73)	6,201 (7.54)	2,372 (8.40)	21,221 (6.91)
Incomplete higher education	257 (0.65)	185 (0.21)	367 (0.28)	476 (0.58)	197 (0.70)	1,482 (0.48)
Complete higher education	552 (1.40)	403 (0.45)	674 (0.51)	875 (1.06)	337 (1.19)	2,841 (0.93)
Not applicable	1,601 (4.07)	3,582 (4.04)	5,391 (4.06)	2,424 (2.95)	910 (3.22)	13,908 (4.53)
Unknown	11,542 (29.36)	30,647 (34.59)	33,265 (25.03)	28,293 (34.41)	6,466 (22.89)	110,213 (35.90)
Total	33,077 (100.00)	70,369 (100.00)	109,156 (100.00)	70,096 (100.00)	24,335 (100.00)	307,033 (100.00)
