# Supplementary material for: Sociodemographic, geographic and temporal patterns of snakebites in Brazil: a descriptive study, 2010-2022
Source: Epidemiol Serv Saude. 2026 May 11;35:e20250181. doi: 10.1590/S2237-96222026v35e20250181.en (PMC13686398; doi:10.1590/S2237-96222026v35e20250181.en)
Supplement: Supplementary file 2 [file 2237-9622-ress-35-e20250181-tabelas-suplementares-pt.pdf]

Tabela Suplementar 1. Características sociodemográficas dos acidentes ofídicos no Brasil entre 2010 e 2022 (n=329.269): análise segundo raça/cor da pele, nível de escolaridade, zona de ocorrência do acidente e intervalo de tempo entre a picada e o atendimento. Os dados são apresentados em número absoluto (N°) e percentual (%) de casos e óbitos de acordo com o tipo de acidente

Avaliação Sociodemográfica										
Raça/cor da pele	Botrópico		Crotálico		Elapídico		Laquético		Total	
	Casos	Óbito	Casos	Óbito	Casos	Óbito	Casos	Óbito	Casos	Óbito
	N° (%)	N° (%)	N° (%)	N° (%)	N° (%)	N° (%)	N° (%)	N° (%)	N° (%)	N° (%)
Parda	163.246 (57,63)	640 (56,99)	17.722 (52,99)	195 (62,10)	2.089 (56,22)	2 (33,33)	6.569 (74,13)	47 (66,20)	189.626 (57,59)	884 (58,39)
Branca	63.502 (22,42)	219 (19,50)	9.107 (27,23)	54 (17,20)	748 (20,13)	1 (16,67)	487 (5,50)	3 (4,23)	73.844 (22,43)	277 (18,30)
Preta	21.460 (7,58)	82 (7,30)	2.680 (8,01)	20 (6,37)	263 (7,08)	0 (0,00)	502 (5,67)	1 (1,41)	24.905 (7,56)	103 (6,80)
Indígena	12.445 (4,39)	96 (8,55)	958 (2,86)	17 (5,41)	81 (2,18)	1 (16,67)	847 (9,56)	18 (25,35)	14.331 (4,35)	132 (8,72)
Amarela	2.310 (0,82)	5 (0,45)	282 (0,84)	2 (0,64)	17 (0,46)	0 (0,00)	48 (0,54)	0 (0,00)	2.657 (0,81)	7 (0,46)
Ignorado	20.282 (7,16)	81 (7,21)	2.698 (8,07)	26 (8,28)	518 (13,94)	2 (33,33)	408 (4,60)	2 (2,82)	23.906 (7,26)	111 (7,33)
Total	283.245 (100,00)	1123 (100,00)	33.447 (100,00)	314 (100,00)	3.716 (100,00)	6 (100,00)	8.861 (100,00)	71 (100,00)	329.269 (100,00)	1.514 (100,00)
**Escolaridade**										
Analfabeto	13.055 (4,61)	117 (10,42)	1.557 (4,66)	27 (8,60)	164 (4,41)	2 (33,33)	513 (5,79)	9 (12,68)	15.289 (4,64)	155 (10,24)
Fundamental I	68.262 (24,10)	306 (27,25)	7.484 (22,38)	81 (25,80)	566 (15,23)	0 (0,00)	2.405 (27,14)	18 (25,35)	78.717 (23,91)	405 (26,75)
Fundamental II	55.736 (19,68)	161 (14,34)	6.229 (18,62)	41 (13,06)	609 (16,39)	0 (0,00)	1.759 (19,85)	12 (16,90)	64.333 (19,54)	214 (14,13)
Ensino médio	33.121 (11,69)	62 (5,52)	4.264 (12,75)	16 (5,10)	472 (12,70)	0 (0,00)	873 (9,85)	3 (4,23)	38.730 (11,76)	81 (5,35)
Ensino superior	3.782 (1,34)	12 (1,07)	490 (1,47)	2 (0,64)	78 (2,10)	0 (0,00)	61 (0,69)	1 (1,41)	4.411 (1,34)	15 (0,99)
Não se aplica	12.095 (4,27)	38 (3,38)	1.512 (4,52)	11 (3,50)	268 (7,21)	0 (0,00)	386 (4,36)	4 (5,63)	14.261 (4,33)	53 (3,50)
Ignorado	97.194 (34,31)	427 (38,02)	11.911 (35,61)	136 (43,31)	1.559 (41,95)	4 (66,67)	2.864 (32,32)	24 (33,80)	113.528 (34,48)	591 (39,04)
Total	283.245 (100,00)	1123 (100,00)	33.447 (100,00)	314 (100,00)	3.716 (100,00)	6 (100,00)	8.861 (100,00)	71 (100,00)	329.269 (100,00)	1.514 (100,00)

Tabela Suplementar 1. Continuação

Raça/cor da pele	Avaliação Sociodemográfica									
	Botrópico		Crotálico		Elapídico		Laquético		Total	
	Casos	Óbito	Casos	Óbito	Casos	Óbito	Casos	Óbito	Casos	Óbito
	Nº (%)	Nº (%)	Nº (%)	Nº (%)	Nº (%)	Nº (%)	Nº (%)	Nº (%)	Nº (%)	Nº (%)
**Zona de Ocorrência e Tempo de acesso ao Tratamento**										
Rural	177.568 (62,69)	724 (64,47)	17.928 (53,60)	183 (58,28)	1.774 (47,74)	4 (66,67)	6.565 (74,09)	56 (78,87)	203.835 (61,91)	967 (63,87)
Urbana	93.311 (32,94)	358 (31,88)	14.123 (42,23)	111 (35,35)	1.732 (46,61)	1 (16,67)	2.004 (22,62)	15 (21,13)	111.170 (33,76)	485 (32,03)
Periurbana	2.664 (0,94)	3 (0,27)	215 (0,64)	0 (0,00)	56 (1,51)	0 (0,00)	56 (0,63)	0 (0,00)	2.991 (0,91)	3 (0,20)
Ignorado	9.702 (3,43)	38 (3,38)	1.181 (3,53)	20 (6,37)	154 (4,14)	1 (16,67)	236 (2,66)	0 (0,00)	11.273 (3,42)	59 (3,90)
Total	283.245 (100,00)	1123 (100,00)	33.447 (100,00)	314 (100,00)	3.716 (100,00)	6 (100,00)	8.861 (100,00)	71 (100,00)	329.269 (100,00)	1.514 (100,00)
**Tempo entre picada de atendimento**										
1 a 3h	95.272 (33,64)	283 (25,20)	10.894 (32,57)	74 (23,57)	1.125 (30,27)	1 (16,67)	2.453 (27,68)	6 (8,45)	109.744 (33,33)	364 (24,04)
0 a 1h	81.505 (28,78)	201 (17,90	11.617 (34,73)	48 (15,29)	1.323 (35,60)	1 (16,67)	1.452 (16,39)	6 (8,45)	95.897 (29,12)	256 (16,91)
3 a 6h	47.938 (16,92)	200 (17,81)	4.807 (14,37)	48 (15,29)	524 (14,10)	3 (50,00)	2.030 (22,91)	10 (14,08)	55.299 (16,79)	261 (17,24)
6 a 12h	19.403 (6,85)	105 (9,35)	1.720 (5,14)	36 (11,46)	191 (5,14)	0 (0,00)	1.112 (12,55)	17 (23,94)	22.426 (6,81)	158 (10,44)
12 a 24h	12.334 (4,35)	111 (9,88)	1.322 (3,95)	42 (13,38)	116 (3,2)	0 (0,00)	818 (9,23)	10 (14,08)	14.590 (4,43)	163 (10,77)
24h ou +	10.894 (3,85)	125 (11,13)	1.070 (3,20)	36 (11,46)	104 (2,80)	0 (0,00)	557 (6,29)	12 (16,90)	12.625 (3,83)	173 (11,43)
Ignorado	15.899 (5,61)	98 (8,73)	2.017 (6,03)	30 (9,55)	333 (8,96)	1 (16,67)	439 (4,95)	10 (14,08)	18.688 (5,68)	139 (9,18)
Total	283.245 (100,00)	1123 (100,00)	33.447 (100,00)	314 (100,00)	3.716 (100,00)	6 (100,00)	8.861 (100,00)	71 (100,00)	329.269 (100,00)	1.514 (100,00)

Tabela suplementar 2. Características sociodemográficas dos acidentes ofídicos no Brasil entre 2010 e 2022 (n=329.269): análise segundo nível de escolaridade e região. Os dados são apresentados em número absoluto e percentual (%)

Escolaridade	Centro-Oeste	Nordeste	Norte	Sudeste	Sul	Total
	N (%)	N (%)	N (%)	N (%)	N (%)	N (%)
Analfabeto	1.148 (2,92)	5.257 (5,93)	6.483 (4,88)	1.637 (1,99)	396 (1,40)	14.921 (4,86)
1ª a 4ª série incompleta do EF	4.805 (12,22)	12.499 (14,11)	23.268 (17,51)	9.823 (11,95)	4.062 (14,38)	54.457 (17,74)
4ª série completa do EF (antigo 1° grau)	2.105 (5,35)	4.721 (5,33)	8.646 (6,51)	5.027 (6,11)	2.364 (8,37)	22.863 (7,45)
5ª à 8ª série incompleta do EF (antigo ginásio ou 1° grau)	5.292 (13,46)	8.843 (9,98)	19.864 (14,95)	8.838 (10,75)	4.334 (15,34)	47.171 (15,36)
Ensino fundamental completo (antigo ginásio ou 1° grau)	1.902 (4,84)	2.893 (3,26)	5.312 (4,00)	4.159 (5,06)	1.798 (6,37)	16.064 (5,23)
Ensino médio incompleto (antigo colegial ou 2° grau)	2.211 (5,52)	3.047 (3,44)	6.080 (4,58)	3.980 (4,84)	1.495 (5,29)	16.813 (5,48)
Ensino médio completo (antigo colegial ou 2° grau)	2.810 (7,15)	3.549 (4,01)	6.289 (4,73)	6.201 (7,54)	2.372 (8,40)	21.221 (6,91)
Educação superior incompleta	257 (0,65)	185 (0,21)	367 (0,28)	476 (0,58)	197 (0,70)	1.482 (0,48)
Educação superior completa	552 (1,40)	403 (0,45)	674 (0,51)	875 (1,06)	337 (1,19)	2.841 (0,93)
Não se aplica	1.601 (4,07)	3.582 (4,04)	5.391 (4,06)	2.424 (2,95)	910 (3,22)	13.908 (4,53)
Ignorado	11.542 (29,36)	30.647 (34,59)	33.265 (25,03)	28.293 (34,41)	6.466 (22,89)	110.213 (35,90)
**Total**	33.077 (100,00)	70.369 (100,00)	109.156 (100,00)	70.096 (100,00)	24.335 (100,00)	307.033 (100,00)
