# Supplementary material for: Sociodemographic, geographic and temporal patterns of snakebites in Brazil: a descriptive study, 2010-2022
Source: Epidemiol Serv Saude. 2026 May 11;35:e20250181. doi: 10.1590/S2237-96222026v35e20250181.en (PMC13686398; doi:10.1590/S2237-96222026v35e20250181.en)
Supplement: Translation

# Translation: Padrões sociodemográficos, geográficos e temporais dos acidentes ofídicos no Brasil: estudo descritivo, 2010-2022

**DOI:** 10.1590/S2237-96222026v35e20250181.pt

**Authors:** Gisele Dias de Freitas, Thiago Salomão de Azevedo, Anderson de Oliveira, Alec Brian Lacerda, Gabriella Pereira dos Santos, Etna Leal, Flávio Santos Dourado, Hui Wen Fan, Francisco Chiaravalloti-Neto

**Affiliations:** ¹ Secretaria da Saúde do Estado de São Paulo, Divisão de Zoonoses, São Paulo, SP, Brasil Secretaria da Saúde do Estado de São Paulo Divisão de Zoonoses São Paulo SP Brasil; 2 Universidade de São Paulo, Faculdade de Saúde Pública, São Paulo, SP, Brasil Universidade de São Paulo Faculdade de Saúde Pública São Paulo SP Brasil; 3 Universidade Estadual Paulista, Instituto de Biociências, Rio Claro, SP, Brasil Universidade Estadual Paulista Instituto de Biociências Rio Claro SP Brasil; 4 Ministério da Saúde, Coordenação-Geral de Vigilância de Zoonoses e Doenças de Transmissão Vetorial, Brasília, DF, Brasil Ministério da Saúde Coordenação-Geral de Vigilância de Zoonoses e Doenças de Transmissão Vetorial Brasília DF Brasil; 5 Instituto Butantan, Centro Bioindustrial, São Paulo, SP, Brasil Instituto Butantan Centro Bioindustrial São Paulo SP Brasil

**Keywords:** Acidentes Ofídicos, Animais Peçonhentos, Antivenenos, Áreas de Risco, Estudo Descritivo.

## Abstract

**Objetivo:**

Analisar os acidentes ofídicos ocorridos no Brasil entre 2010 e 2022 com foco sobre: características sociodemográficas das vítimas, gênero das serpentes envolvidas, sazonalidade, distribuição geográfica, grau de risco e relação com os biomas, através de um estudo descritivo baseado em dados do Sistema de Informação de Agravos de Notificação sobre acidentes causados por *Bothrops, Crotalus, Lachesis* e *Elapidae*.

**Métodos:**

Foram calculadas taxas de incidência, mortalidade e letalidade por gênero de serpente, idade e sexo. A análise espacial utilizou estatísticas bayesianas e o índice de Getis-Ord (Gi*) para identificar áreas de maior concentração de casos e classificar o risco regional (de muito baixo a muito alto).

**Resultados:**

Entre 2010 e 2022, foram registrados 329.269 acidentes ofídicos no país, com predominância dos casos botrópicos (70,6%). No mesmo período, ocorreram 1.514 óbitos, sendo 74,2% associados a acidentes botrópicos, 20,7% a crotálicos, 4,7% a laquéticos e 0,4% a elapídicos. Apesar da menor frequência, os acidentes crotálicos apresentaram a maior letalidade anual (0,1%). A maioria das vítimas eram homens (77,8%), pardos (58,4%), adultos (76,3%), com baixa escolaridade (48,0%) e residentes em áreas rurais (63,9%). Óbitos foram associados a atrasos no atendimento. Os acidentes botrópicos permaneceram estáveis, os crotálicos e elapídicos aumentaram, enquanto os laquéticos diminuíram. Os casos ocorreram majoritariamente entre outubro e março, exceto no Nordeste, entre dezembro e junho. Geograficamente, os padrões seguiram os biomas.

**Conclusão:**

Os resultados reforçam a necessidade de estratégias regionais específicas para vigilância, prevenção e manejo eficaz, fundamentadas nos padrões geográficos, sazonais e demográficos observados.

**Ethical aspects**

Esta pesquisa utilizou bancos de dados de domínio público e anonimizados.

## Introdução 

O envenenamento por picadas de serpentes é uma doença tropical negligenciada que resulta em aproximadamente 138 mil mortes anuais no mundo, além de causar sofrimento e incapacidades, especialmente em comunidades pobres de países de baixa e média renda ^1^. Reconhecendo a gravidade, em 2017, a Organização Mundial da Saúde incluiu o envenenamento por picada de cobra na lista de doenças tropicais negligenciadas e, em 2019, aprovou a *Estratégia para uma resposta globalmente coordenada a uma doença tropical negligenciada prioritária*, na qual estabeleceu a meta de reduzir em 50% as mortes e as incapacidades relacionadas ao ofidismo até 2030 através de esforços globais. As principais estratégias são: (i) melhorar a vigilância do agravo; (ii) aumentar o conhecimento epidemiológico dos envenenamentos, para identificar e atender de forma mais eficaz as populações que necessitam de maior educação preventiva, tratamento adequado e assistência a longo prazo devido a sequelas; e (iii)ampliar a disponibilidade, a acessibilidade e a redução do custo dos antivenenos em nível global, para garantir tratamentos farmacoterapêuticos seguros e eficazes para os acidentados ^2^^,^^3^. 

No Brasil, pela vasta biodiversidade, os acidentes com animais peçonhentos têm grande relevância à saúde pública. No período analisado, o país ocupava o terceiro lugar no mundo em número de acidentes ofídicos, ao lado do Vietnã, ficando atrás da Índia e do Sri Lanka ^4^^,^^5^. Em 2023, foram registrados 32.595 acidentes e 143 óbitos, principalmente nas regiões Norte e Nordeste, que juntas notificaram quase dois terços dos casos, apesar de abrigarem apenas 36% da população brasileira. Esses acidentes são agravados por fatores como a invasão de habitats naturais e o acúmulo de lixo e de entulho, que atraem pequenos animais, alimento para as serpentes ^6^. 

Desde a criação do soro antiofídico por Vital Brazil e a fundação do Instituto Butantan, em 1901 ^7^, até a implementação do Programa Nacional de Ofidismo, em 1986, após uma grande crise na produção de soro no país, que culminou com a morte de uma criança em Brasília ^8^, o Brasil tem investido no controle de acidentes por animais peçonhentos. As notificações desses acidentes são realizadas no Sistema de Informação de Agravos de Notificação (Sinan) e ganharam maior representatividade, especialmente a partir de 2010, após a inclusão do agravo na lista de notificações obrigatórias ^9^. À época, essas informações forneceram dados fundamentais para a vigilância epidemiológica e o planejamento de ações de saúde pública.

No país, as serpentes das famílias Viperidae (jararacas, cascavéis e surucucus) e Elapidae (corais) são responsáveis pelos acidentes botrópicos, crotálicos, laquéticos e elapídicos, respectivamente ^10^. Dentre elas, o gênero *Bothrops* responde pela maioria dos acidentes, enquanto o *Crotalus* apresenta a maior letalidade ^11^.

O tratamento do envenenamento após acidente exige a aplicação de antiveneno (soro) específico para cada tipo de acidente. Esses soros são fornecidos exclusivamente pelo Ministério da Saúde, fazem parte do Componente Estratégico da Assistência Farmacêutica e estão incluídos na Relação Nacional de Medicamentos Essenciais. Esses imunobiológicos só são disponibilizados gratuitamente em unidades de saúde públicas, que são pontos estratégicos designados pelo Sistema Único de Saúde para atender a população. No *Guia de Vigilância em Saúde* do Ministério da Saúde, estão descritos os protocolos de tratamento, incluindo o número de frascos a serem utilizados conforme a gravidade do acidente (leve, moderado ou grave) ^12^.

Os antivenenos são produtos biológicos obtidos a partir do plasma processado e purificado de equinos previamente imunizados com antígenos de venenos animais. Seu processo de fabricação é demorado e depende da resposta imunológica dos animais, tornando seu uso restrito a casos realmente necessários. Para minimizar complicações e reduzir o risco de sequelas, o tratamento deve ser administrado o mais rápido possível, idealmente dentro das primeiras seis horas após a picada ^12^.

Diante desse cenário, o principal objetivo deste estudo foi analisar os acidentes ofídicos ocorridos no Brasil entre 2010 e 2022 com foco nas características sociodemográficas das vítimas, no gênero das serpentes envolvidas, na sazonalidade, na distribuição geográfica, no grau de risco e na relação com os biomas.

Os resultados obtidos têm o objetivo de contribuir para a identificação de áreas de maior risco, subsidiando estratégias de prevenção, a alocação de recursos em regiões prioritárias e o planejamento adequado do atendimento e da distribuição de soros, conforme o tipo de acidente e de padrões regionais.

## Métodos 

Tipo de estudo e fonte de dados

Tratou-se de estudo descritivo sobre acidentes ofídicos ocorridos no Brasil entre 2010 e 2022. Os dados foram extraídos do Sinan, sendo selecionadas todas as notificações de acidentes com animais peçonhentos que envolviam serpentes dos gêneros *Bothrops*, *Crotalus*, *Lachesis* e da família *Elapidae*. Foram incluídos apenas os registros com dados completos sobre o tipo de serpente e a localização do acidente. Considerou-se como acidente autóctone aquele em que o município de residência do paciente coincidiu com o município de ocorrência.

Contextualização

As variáveis analisadas incluíram características sociodemográficas dos acidentados (idade, sexo, raça/cor da pele e escolaridade), além de informações espaço-temporais (data do acidente, município de residência e ocorrência, zona de ocorrência) e variáveis relacionadas ao atendimento e ao desfecho do caso (tempo entre acidente e atendimento, bem como evolução clínica). As informações demográficas e geográficas adicionais foram obtidas junto ao Instituto Brasileiro de Geografia e Estatística, incluindo dados sobre os biomas nacionais (Amazônia, Caatinga, Cerrado, Mata Atlântica, Pampa e Pantanal) ^13^.

Análise estatística

Foram calculadas as taxas de incidência, mortalidade e letalidade por gênero de serpente, faixa etária (0-9 anos, 10-19 anos, 20-59 anos e ≥60 anos) e sexo. A taxa de incidência anual foi obtida dividindo-se o número médio de acidentes pela população estimada para 2016 dividida por 13 (número de anos do estudo), multiplicando-se por 100 mil habitantes. A taxa de mortalidade foi calculada da mesma forma, substituindo-se o número de acidentes pelo número de óbitos. A letalidade foi determinada pela razão entre o número de óbitos e o número total de acidentes, multiplicada por 100 mil habitantes.

Para a análise temporal, foi avaliado o número de acidentes segundo o gênero da serpente, a região geográfica, o ano de ocorrência (temporalidade) e o mês de ocorrência (sazonalidade). A distribuição espacial das taxas de incidência foi analisada por meio de taxas ajustadas por estimativas bayesianas empíricas locais ^14^, considerando apenas os acidentes autóctones por tipo de serpente e por município de ocorrência. Para identificar áreas de concentração de casos, aplicou-se o índice de Getis-Ord (Gi*), capaz de detectar aglomerados espaciais com valores acima ou abaixo da média regional, além de quantificar discrepâncias ^15^.

A contagem anual de ocorrências foi normalizada em escores Z e organizada anualmente pelo valor máximo dentro do limite geográfico ^16^. A estratificação de risco foi realizada com base na frequência percentual dos municípios identificados como áreas de concentração de casos ao longo do período de estudo, sendo classificados em cinco níveis: muito baixo (≤20%), baixo (20%-40%), médio (40%-60%), alto (60%-80%) e muito alto (>80%). As análises foram realizadas com o auxílio dos softwares Excel, GeoDa, QGIS, R (versão 4.3.2) e Tabwin, e os resultados foram apresentados por meio de mapas qualitativos separados por gênero de serpente, cujo acesso à base de dados encontra-se na plataforma Zonedo ^17^.

## Resultados 

Entre 2010 e 2022, no Brasil, foram notificados 329.269 acidentes causados por serpentes peçonhentas. Desse total, 283.245 (70,6%) foram botrópicos, 33.447 (8,3%) crotálicos, 8.861 (2,2%) laquéticos e 3.716 (0,9%) elapídicos. No mesmo período, foram registrados 1.514 óbitos relacionados a esses acidentes, sendo 1.123 (74,2%) atribuídos a acidentes botrópicos, 314 (20,7%) a crotálicos, 71 (4,7%) a laquéticos e 6 (0,4%) a elapídicos. A análise da autoctonia dos acidentes revelou que uma parcela dos casos ocorreu fora do município de residência: 7,3% nos botrópicos, 9,6% nos crotálicos, 7,7% nos elapídicos e 5,0% nos laquéticos.

A raça/cor da pele parda foi a mais prevalente nos acidentes e óbitos, mas observou-se uma alta proporção de indígenas entre os acidentes laquéticos (9,6% dos acidentes e 25,4% dos óbitos). Os acidentes ofídicos foram mais frequentes entre homens (77,8%), pardos (58,4%), adultos entre 20-59 anos (76,3%) e pessoas com escolaridade até o ensino fundamental (48,0%). A maioria ocorreu em zonas rurais (63,9%), embora os acidentes crotálicos e os elapídicos tenham apresentado proporções relevantes em áreas urbanas (42,2% e 46,6%). Quanto ao tempo de atendimento, 65,3% das vítimas de acidentes botrópicos, crotálicos e elapídicos foram atendidas em até três horas, enquanto nos acidentes laquéticos esse percentual foi menor (44,1%), com 35,4% recebendo atendimento entre três e 12 horas (Tabela suplementar 1).

Ainda em relação à avaliação sociodemográfica, a análise da escolaridade das vítimas de acidentes ofídicos, segundo as regiões do Brasil, revelou uma predominância de indivíduos com baixa escolaridade, especialmente nas regiões Norte, Nordeste e Centro-Oeste. O analfabetismo era mais prevalente nas regiões Norte e Nordeste, enquanto as regiões Sul e Sudeste apresentaram menores proporções de analfabetismo. A presença de escolaridade superior (completa ou incompleta) foi reduzida em todas as regiões. Destacou-se, contudo, o elevado percentual de registros com escolaridade ignorada, com 28,4% na região Norte, 40,7% na Nordeste, 33,2% na Centro-Oeste, 26,6% na Sul e 39,4% na Sudeste, o que comprometeu a qualidade dos dados e limitou análises mais precisas (Tabela suplementar 2). 

Nos óbitos, o perfil socioeconômico foi semelhante ao dos acidentados, mas houve maior proporção de vítimas indígenas nos casos elapídicos (16,7%) e laquéticos (25,4%). A baixa escolaridade (51,1%) foi mais comum entre os óbitos, e a predominância dos acidentes em zonas rurais foi ainda mais acentuada (78,9%). O tempo para atendimento foi significativamente maior nos casos fatais, especialmente nos acidentes laquéticos.

Os acidentes botrópicos foram os mais frequentes no Brasil, com incidência 7,4 vezes maior que os crotálicos, 77,5 vezes maior que os elapídicos e 30,9 vezes maior que os laquéticos. Homens foram os mais afetados em todos os tipos de acidentes, com destaque para os laquéticos e os crotálicos, nos quais a incidência foi três vezes maior do que em mulheres, enquanto nos acidentes elapídicos essa diferença foi 1,5 vez. Os acidentes crotálicos apresentaram a maior letalidade, seguidos pelos laquéticos, pelos botrópicos e pelos elapídicos. Idosos foram os mais propensos a óbito após acidente ofídico. A letalidade variou regionalmente, sendo maior nas regiões Norte e Centro-Oeste para os acidentes botrópicos, crotálicos e laquéticos, e, na região Sul, para os crotálicos e elapídicos. Por faixa etária, os acidentes botrópicos foram mais comuns entre 10-19 anos e 20-59 anos, com 12,0 e 11,4 casos por 100 mil habitantes ao ano. Os acidentes crotálicos afetaram mais adultos e idosos, enquanto os elapídicos e laquéticos seguiram o padrão dos botrópicos (Tabela 1).

**Tabela 1:** Análise dos acidentes ofídicos por sexo, faixa etária, região e tipo de acidente, segundo o número de acidentes e óbitos e as taxas de incidência (100 mil habitantes/ano), mortalidade (100 mil habitantes/ano) e letalidade (%). Brasil, 2010-2022 (n=329.269)

Tipo de acidente	Variável	Sexo^a^		Faixa etária (anos)				Região do Brasil^b^					Total
		F	M	0-9	10-19	20-59	≥60	N	NE	CO	SE	S	
Botrópico	Número de acidentes	63.511	219.734	20.697	47.069	180.862	34.617	106.926	63.210	30.223	59.619	23.267	283.245
	Taxa de incidência	4,48	16,21	5,42	12,00	11,36	8,50	43,50	8,43	13,92	5,12	5,89	10,21
	Número de óbitos	309	814	65	82	571	405	414	328	147	166	68	1.123
	Taxa de mortalidade	2,18	6,01	1,70	2,09	3,59	9,94	16,84	4,38	6,77	1,42	1,72	4,05
	Letalidade	0,37	0,28	0,24	0,13	0,24	0,90	0,03	0,04	0,04	0,02	0,02	0,03
Crotálico	Número de acidentes	6.825	26.622	2.402	4.450	21.707	4.888	2.949	12.535	4.580	11.797	1.586	33.447
	Taxa de incidência	0,48	1,96	0,63	1,13	1,36	1,20	1,20	1,67	2,11	1,01	0,40	1,21
	Número de óbitos	64	250	21	25	149	119	38	163	31	68	14	314
	Taxa de mortalidade	0,45	1,84	0,55	0,64	0,94	2,92	1,55	2,17	1,43	0,58	0,35	1,13
	Letalidade	0,72	0,72	0,67	0,43	0,53	1,87	0,10	0,10	0,05	0,04	0,07	0,07
Laquético	Número de acidentes	1.666	7.195	754	1.849	5.495	763	7.956	495	291	101	18	8.861
	Taxa de incidência	0,12	0,53	0,20	0,47	0,35	0,19	3,24	0,07	0,13	0,01	0,00	0,32
	Número de óbitos	13	58	11	6	35	19	68	0	3	0	0	71
	Taxa de mortalidade	0,09	0,43	0,29	0,15	0,22	0,47	2,77	0,00	0,14	0,00	0,00	0,06
	Letalidade	0,60	0,62	1,12	0,25	0,49	1,92	0,07	0,00	0,08	0,00	0,00	0,06
Elapídico	Número de acidentes	1.080	2.636	353	530	2.483	350	437	2.043	270	741	225	3.716
	Taxa de incidência	0,08	0,19	0,09	0,14	0,16	0,09	0,18	0,27	0,12	0,06	0,06	0,13
	Número de óbitos	1	5	0	1	3	2	1	3	0	1	1	6
	Taxa de mortalidade	0,01	0,04	0,00	0,03	0,02	0,05	0,04	0,04	0,00	0,01	0,03	0,02
	Letalidade	0,07	0,15	0,00	0,15	0,09	0,44	0,02	0,01	0,00	0,01	0,03	0,01
Total	Número de acidentes	73.082	256.187	24.206	53.898	210.547	40.618	118.268	78.283	35.364	72.258	25.096	329.269
	Taxa de incidência	5,51	20,12	6,25	12,23	14,32	13,44	48,12	10,44	16,28	6,20	6,35	12,66
	Número de óbitos	387	1.127	97	114	758	545	521	494	181	235	83	1.514
	Taxa de mortalidade	0,03	0,09	0,03	0,03	0,05	0,18	0,21	0,07	0,08	0,02	0,02	0,06
	Letalidade	0,53	0,44	0,40	0,21	0,36	1,34	0,44	0,63	0,51	0,33	0,33	0,46

a Sexo: F=feminino, M=masculino; ^b^Região: N=Norte, NE=Nordeste, CO=Centro-Oeste, SE=Sudeste, S=Sul.

Quanto à distribuição regional, os acidentes botrópicos foram mais prevalentes na região Norte e menos prevalente na Sudeste. Os crotálicos tiveram maior incidência na região Centro-Oeste e menor na Sul. Os acidentes elapídicos foram mais frequentes na região Nordeste e menos, de forma similar, nas regiões Sul e Sudeste. Já os laquéticos predominaram na região Norte e foram menos frequentes na Sudeste. Na região Sul, não era esperada a notificação de acidentes laquéticos, pois não há registro de serpentes desse gênero em vida livre na área (Tabela 1) (18).

No período avaliado, os acidentes ofídicos apresentaram diferentes tendências temporais. Os acidentes botrópicos mantiveram um padrão de ocorrência estável na região Centro-Oeste, tendência de elevação no número de acidentes nas regiões Norte e Nordeste e de diminuição nas regiões Sul e Sudeste. Os acidentes crotálicos e elapídicos demonstraram uma tendência de aumento, a partir de 2013, nas regiões Sul e Nordeste e de estabilidade nas outras. Já os acidentes laquéticos apresentaram uma redução acentuada e contínua no número de casos na região Norte desde 2010 (Figuras 2A a 2D).

**Figura 1:** Geoespacialidade da América do Sul com detalhamento do território brasileiro: (A) regiões brasileiras; (B) biomas e unidades federativas (UF)

**Figura 2:** Análises temporal (A-D) e sazonal (E-H) dos acidentes ofídicos estratificadas por tipo de acidente e região. Brasil, 2010-2022 (n=329.269)

Na análise da sazonalidade, considerando o mês de ocorrência, houve padrões regionais distintos. Nos acidentes botrópicos, as regiões Centro-Oeste, Norte, Sudeste e Sul apresentaram maior frequência entre outubro e março, enquanto na região Nordeste o período de maior ocorrência foi entre fevereiro e junho, em sentido oposto às demais regiões. Nos acidentes crotálicos, as regiões Centro-Oeste, Sudeste e Sul seguiram um padrão semelhante, com maior número de casos entre novembro e abril, enquanto nas regiões Norte e Nordeste a sazonalidade foi mais acentuada entre dezembro e junho. Os acidentes elapídicos mostraram um padrão uniforme entre as regiões, com maior ocorrência de casos entre janeiro e maio. Já nos acidentes laquéticos, destacou-se a região Norte, com aumento dos casos a partir de dezembro, atingindo o pico em março e registrando o menor número de ocorrências entre agosto e setembro (Figuras 2E a 2H).

Independentemente do gênero de serpente causadora do acidente, houve uma grande correlação entre os padrões espaciais de distribuição das taxas anuais de incidência, os locais de maior risco e os locais onde ocorreram os óbitos após os referidos acidentes (Figuras 3 e 4).

**Figura 3:** Distribuição espacial dos acidentes botrópicos (n=283.245) (A-C) e laquéticos (n=8.861) (D-F) por municípios e estados estratificada por taxas de incidência bayesiana^a^ (A e D), áreas de risco (B e E) e número de óbitos (C e F). Brasil, 2010-2022

Os acidentes botrópicos foram amplamente distribuídos por todo o território brasileiro, porém, conforme mostrado na Figura 3A, e podendo relacionar à distribuição dos biomas na Figura 1, as regiões com as maiores taxas anuais de incidência dos acidentes botrópicos encontravam-se nos biomas Amazônia, Mata Atlântica e em parte do bioma Cerrado, o que coincidiu também com o mapa de risco para esse tipo de acidente e com a maior ocorrência de óbitos (Figuras 3A a 3C). Os acidentes laquéticos apresentaram um padrão de distribuição mais característico e restrito, sendo mais frequente na região Norte e no extremo do bioma Mata Atlântica, entre os estados da região Nordeste e o Espírito Santo (Figura 3D). Já os acidentes crotálicos apresentaram padrão de distribuição onde há predomínio do bioma Cerrado e nos estados que ficam no extremo norte do bioma Amazônia (Amapá e Roraima) (Figura 4A). Por fim, os acidentes elapídicos apresentaram locais com maior risco de acidentes nos biomas Amazônia, Cerrado, Caatinga e Mata Atlântica (Figura 4D).

**Figura 4:** Distribuição espacial dos acidentes crotálicos (n=33.447) (A-C) e elapídicos (n=3.716) (D-F) por municípios e estados estratificada por taxas de incidência bayesiana^a^ (A e D), áreas de risco (B e E) e número de óbitos (C e F). Brasil, 2010-2022.

## Discussão 

Entre 2010 e 2022, os acidentes ofídicos no Brasil mostraram padrões marcantes em relação à distribuição geográfica, ao perfil das vítimas e às características clínicas. Observou-se o predomínio de acidentes botrópicos, responsáveis por dois terços dos casos e dos óbitos, o que pode refletir a ampla distribuição e a adaptabilidade das serpentes do gênero *Bothrops*. Apesar de menos frequentes, os acidentes crotálicos apresentaram maior letalidade, evidenciando sua gravidade clínica. A maioria das vítimas era composta por homens adultos, pardos, com baixa escolaridade e residentes em zonas rurais, indicando uma relação entre vulnerabilidade socioeconômica, exposição ocupacional e dificuldades no acesso ao atendimento. Notou-se ainda que acidentes laquéticos, embora em declínio, seguem sendo relevantes devido à alta letalidade e à ocorrência em regiões remotas, como a Amazônia. A sazonalidade dos casos variou regionalmente, com maior concentração no período chuvoso, sugerindo a influência de fatores ecológicos e climáticos sobre a atividade das serpentes e o risco de acidentes.

Entre as limitações do estudo, destaca-se a dependência de dados secundários provenientes do Sinan, os quais estão sujeitos a subnotificação, inconsistências e preenchimento incompleto. Além disso, erros na identificação das serpentes ou no tipo de envenenamento podem gerar distorções nos dados, como a possível superestimação dos acidentes laquéticos em decorrência da nomenclatura popular indistinta em algumas regiões. Outra limitação refere-se à ausência de dados detalhados sobre o local de ocorrência dos óbitos e à diferenciação entre áreas urbanas e rurais, o que compromete análises mais refinadas sobre os fatores de risco e o acesso aos serviços de saúde. Além disso, a baixa completitude das variáveis relacionadas ao nível de escolaridade dos acidentados limita a análise mais detalhada do perfil sociodemográfico das vítimas.

A alta frequência de acidentes botrópicos está de acordo com estudos anteriores que destacam a ampla distribuição das serpentes do gênero *Bothrops* em todos os biomas brasileiros, desde florestas densas até áreas antropizadas ^11^^,^^19^. Essa plasticidade ecológica favorece o contato com humanos em diferentes contextos. Já a elevada letalidade dos acidentes crotálicos pode ser atribuída à toxicidade do veneno e à rápida progressão dos sintomas clínicos, exigindo diagnóstico precoce e administração imediata de soro antiofídico, como observado também em estudo de coorte retrospectivo na região Centro-Oeste ^20^. Quanto aos acidentes laquéticos, além da gravidade do envenenamento, fatores geográficos e culturais dificultam o acesso ao atendimento, especialmente entre populações indígenas que, por vezes, recorrem inicialmente à medicina tradicional. Essa barreira cultural foi igualmente observada em pesquisa qualitativa realizada com comunidades amazônicas ^21^^,^^22^.

O perfil sociodemográfico das vítimas é consistente com achados de outras pesquisas com delineamento transversal realizadas em zonas rurais das regiões Nordeste e Norte do país, que apontam a predominância de acidentes entre adultos jovens, trabalhadores rurais e homens ^9^^,^^23^. Isso sugere que atividades econômicas ao ar livre estão diretamente relacionadas à exposição ao risco. Já a maior frequência de óbitos entre indígenas, especialmente por acidentes laquéticos, pode ser explicada tanto pelo isolamento geográfico quanto pela limitação de recursos nos serviços locais de saúde, como já descrito em investigações na Amazônia Legal ^24^.

A análise temporal revelou a estabilidade dos acidentes botrópicos, provavelmente associada à ampla distribuição do gênero e à constância do contato com humanos. Em contrapartida, o aumento dos acidentes crotálicos pode estar relacionado à expansão dessas espécies em áreas degradadas, conforme descrito em estudos sobre alterações no uso do solo e mudanças climáticas ^11^^,^^19^^,^^25^. A redução dos casos laquéticos, por sua vez, pode decorrer da retração dos habitats das serpentes e da melhoria nos sistemas de notificação. A sazonalidade observada coincide com o período de maior atividade das serpentes e aumento de práticas agrícolas ^26^^,^^27^.

A análise espacial apontou maior letalidade nos acidentes registrados nas regiões Norte e Centro-Oeste, coerente com estudos anteriores que associam mortalidade ao tempo prolongado entre o acidente e o atendimento médico, reflexo da baixa cobertura de serviços de saúde em regiões remotas ^23^. A presença de notificações de acidentes laquéticos fora da área de ocorrência da espécie reforça a hipótese de erro de identificação ou a confusão de nomenclatura regional, como ocorre no Alto Juruá, onde *Bothrops atrox* é chamada de "surucucu", o mesmo nome dado a *Lachesis muta*^28^^,^^29^. Tais erros comprometem a precisão dos dados e exigem investimentos em educação e em capacitação dos profissionais responsáveis pela notificação.

A ocorrência de acidentes não autóctones reforça a mobilidade das vítimas e os desafios logísticos para o atendimento rápido. Estratégias como a ferramenta online do estado de São Paulo para localização de unidades com soro antiofídico demonstram potencial para mitigar atrasos e devem ser replicadas em outras regiões ^30^. A padronização da nomenclatura regional e a capacitação dos profissionais de saúde podem contribuir significativamente para a melhoria da qualidade das notificações e da resposta assistencial.

Em síntese, os achados deste estudo revelam a complexidade dos acidentes ofídicos no Brasil, fortemente condicionados por fatores ecológicos, climáticos, geográficos e sociais. A predominância de acidentes botrópicos, a elevada letalidade de crotálicos e laquéticos e a desigualdade no acesso aos serviços de saúde evidenciam a necessidade de estratégias regionais específicas para vigilância, prevenção e manejo quanto aos acidentes ofídicos. Além disso, destaca-se a importância de ações voltadas ao aprimoramento da qualidade dos dados registrados no Sinan, a fim de permitir análises mais consistentes e aprofundadas sobre o perfil das vítimas. Os resultados obtidos neste estudo oferecem subsídios para políticas públicas mais eficazes e equitativas, especialmente voltadas para populações vulneráveis e para regiões de difícil acesso, promovendo uma abordagem integrada entre saúde, meio ambiente e manejo animal.

## Data Availability

O banco de dados, os códigos, os métodos e outros materiais utilizados e resultantes da pesquisa utilizados neste estudo estão disponíveis para consulta na plataforma Zenodo. Disponível em: Freitas GD de, Oliveira A de. Acidentes ofídicos no Brasil (2010-2022): um retrato nacional dos padrões sociodemográficos, geográficos e temporais em estudo descritivo [Internet]. Zenodo; 2025. Available from: https://doi.org/10.5281/zenodo.15571022.

## Material Suplementar PT

Tabelas Suplementares
